# Radicular Pathology in Zirconia and Stainless-Steel Crowns in Primary Posterior Teeth: A Retrospective Comparative Longitudinal Study

**DOI:** 10.3390/children12101417

**Published:** 2025-10-20

**Authors:** Diana Ram, Maayan Sherman, David Polak, Esti Davidovich

**Affiliations:** 1 Department of Pediatric Dentistry, Hadassah School of Dental Medicine, Hebrew University, Jerusalem 91120, Israel; dianar@ekmd.huji.ac.il (D.R.); maayan.sherman@mail.huji.ac.il (M.S.); 2 Oral Medicine Unit, Sheba Medical Center, Tel-Hashomer, Ramat-Gan 52621, Israel; 3 The Goldschleger School of Dental Medicine, Faculty of Medical and Health Sciences, Tel Aviv University, Tel Aviv 6934228, Israel

**Keywords:** stainless steel crowns, pediatric, zirconia crowns, radicular pathology, primary molar, pulp therapy

## Abstract

Background: Zirconia crowns (ZCs) are increasingly used in pediatric dentistry for their esthetic advantages, but evidence regarding their long-term outcomes compared with stainless steel crowns (SSCs) is limited. Methods: This retrospective cohort study evaluated 138 primary molars in 89 children (mean age: 5.1 ± 1.6 years) restored with ZCs (*n* = 69) or SSCs (*n* = 69). Radiographic and clinical follow-up averaged 27.7 months. Outcomes included radicular pathology, crown retention, and survival. Statistical analyses examined associations with patient age, tooth type, and pulp therapy. Results: Both groups demonstrated high overall success (87%). However, pathology developed more frequently in ZCs compared with SSCs (18% vs. 9%, *p* = 0.0025). Loss of retention was also higher in ZCs (16% vs. 3%, *p* = 0.009). No significant associations were found between pathology and patient age, tooth type, or pulp therapy. SSCs demonstrated longer survival (29.1 vs. 25.1 months, *p* = 0.034) and later onset of pathology (29.1 vs. 12.5 months, *p* = 0.003). Crowns offer an esthetic alternative for restoring primary molars but are associated with higher rates of radicular pathology and retention loss. Careful case selection, optimal isolation, and appropriate cementation are essential for clinical success. Stainless steel crowns remain the more durable option, particularly in complex clinical cases.

## 1. Introduction

Stainless steel crowns (SSCs) are considered the gold standard for the restoration of extensively decayed or structurally compromised primary molars due to their durability and predictable long-term outcomes [1,2]. They are particularly useful after pulp therapy, in multi-surface lesions, and in cases where other restorative options are unlikely to provide sufficient strength. SSCs preserve masticatory function, maintain space for permanent successors, and protect compromised teeth from fracture under occlusal forces. Nevertheless, despite their reliability, SSCs are often regarded as esthetically unacceptable by both parents and patients, especially when placed in visible areas of the mouth.

In response to these esthetic concerns, zirconia crowns (ZCs) have been introduced as a full-coverage restorative option for children. ZCs are tooth-colored, biocompatible, and demonstrate favorable gingival responses compared with metal crowns [3]. Their highly polished surfaces reduce plaque accumulation, and their natural appearance makes them increasingly popular among families seeking esthetic solutions for their children [4,5]. However, ZCs are less forgiving than SSCs: they require more aggressive tooth reduction, precise tooth preparation, and are cemented passively with luting cements, which makes them more technique-sensitive.

The clinical performance of ZCs in primary molars has been reported in short-term studies, often emphasizing esthetics and gingival health. Several systematic reviews and meta-analyses have described encouraging results, noting lower plaque accumulation and improved periodontal indices with ZCs compared with SSCs [4,5]. Yet, despite these findings, there remains considerable heterogeneity among studies, and methodological quality has often been limited [1,6]. Importantly, most available literature focuses on outcomes such as retention or gingival status over 6–12 months, with few studies providing evidence beyond the short-term.

One of the least explored areas concerns the long-term radicular health of primary molars restored with ZCs. While SSCs have decades of data supporting their safety and effectiveness, ZCs lack robust evidence on whether their preparation and cementation protocols may predispose to pulpal or radicular complications. Given that many failures in pediatric dentistry are linked not only to restoration loss but also to the development of pathology, this gap is clinically significant.

Therefore, the aim of the present retrospective study was to compare the long-term outcomes of zirconia and stainless steel crowns in primary molars of children, focusing on radicular pathology, crown retention, and survival.

## 2. Materials and Methods

The study included primary molars that were treated with crowns between January 2015 and January 2020. The study sample (*n* = 138 teeth) included all eligible cases meeting the inclusion criteria during this period. A retrospective power calculation confirmed that this sample size provided 80% power to detect a 10% difference in pathology rates between groups, at α = 0.05. The treatments were performed in three pediatric dental clinics: the Department of Pediatric Dentistry at the Faculty of Dental Medicine of the Hebrew University and Hadassah, and two private pediatric dentistry specialist clinics in Jerusalem and Tel Aviv. The study was conducted according to the guidelines of the Declaration of Helsinki and approved by the Institutional Human Subjects Ethics Committee. All the procedures were performed in accordance with the ethical standards of the institutional and national research committee (Reference number HMO-0411-20). All treatments were performed by two experienced and equally qualified specialists in pediatric dentistry (ED and DR) all of them in the dental chair (either with or without conscious sedation), none of them under general anesthesia. Postoperative radiographs were evaluated by a graduate student (MS) as part of a DMD thesis, which had been pre-planned and supervised by the same two specialists. A calibration process was conducted separately from the study, in which 20 radiographs were assessed to diagnose radicular pathology in primary teeth pre- and postoperatively. The inter-examiner reliability achieved a kappa value of 0.93. We identified 69 children treated with ZCs, and matched them to 69 children treated with SSC, according to age and follow-up period records (measured in months). All the crowns were cemented with glass ionomer cement. Case selection included all zirconia crown (ZC) treatments with sufficient documentation available in the dental records. Stainless steel crown (SSC) cases were matched based on patient age and follow up period.

All SSCs were manufactured by 3M^®^ and placed according to the manufacturer’s clinical guidelines for conventional preparation (no Hall technique was used) Similarly, all ZCs were from the NuSmile^®^ brand, and their placement also adhered strictly to the manufacturer’s instructions.

The choice of crown type was determined based on parental preference. Healthy children of both genders were included, aged 3–9 years at the time of treatment. Additional inclusion criteria were: follow-up of 12 months or more from the end of treatment, and sufficient information in the dental records regarding the treatment (preoperative X-rays, clinical preoperative information, date of treatment, follow-up X-rays and clinical examination).

Data collected from the dental files included: age, patient dental history, the specific teeth treated (#54, #55, #64, #65, #74, #75, #84, #85), the crown type (ZC or SSC) and the date of the crown procedure. The date of the last follow-up visit and follow-up data were recorded.

Data extracted from the children’s dental records included the presence of radiographic pathology, specifically internal or external root resorption and interradicular radiolucency. Additional inclusion criteria were: follow-up of 12 months or more from the end of treatment, and sufficient information in the dental records regarding the treatment (preoperative radiographs, clinical preoperative information, date of treatment, follow-up radiographs, and clinical examination). Exclusion criteria were incomplete dental records, follow-up shorter than 12 months, and preoperative evidence of advanced pathology (such as severe root resorption or periapical lesions) [7].

Treatments were evaluated according to the most updated postoperative radiographs and according to the clinical records in the patients’ files. The follow-up radiographs were performed as part of the routine follow-up. Radiographs were obtained every 12–18 months as part of routine care. Time-to-pathology was defined as the first follow-up visit showing radiographic/clinical pathology.

Treatment was considered successful if at least one of the following criteria was met: absence of radiographic evidence of pathological changes or disease progression, the tooth remained asymptomatic, the crown was retained, or the tooth exfoliated naturally. Treatment was deemed a failure if any of the following conditions were observed: radiographic evidence of resorption or other pathology, dental abcess, extraction of the tooth, or the need for additional intervention such as re-cementation or extraction.

## 3. Statistical Analysis

We compared variables between children who were treated with ZCs and SCCs. These included: the primary molar treated (first or second, upper or lower, the child’s age, the type of crown), whether pulp treatment was performed and the type of pulp treatment, the time until the development of pathology (resorption, abscess or loss of the crown), and survival of the crown. Categorical variables were compared by a Chi squared test (X2). For small subgroup analyses, Fisher’s test was used. Quantitative variables were compared by the Pearson test. Relations between categorical variables and quantitative variables were determined by the *t*-test and by a one-way Anova test. Significance was determined as *p* < 0.05. Normality was tested using Shapiro–Wilk test; homogeneity of variances was confirmed using Levene’s test. Results of these tests supported the use of parametric statistics.

## 4. Results

The study included 69 molars treated with an esthetic ZC, which were matched as described above to 69 molars treated with SCC. The molars were in 89 children. The mean follow-up time was: 27.5 ± 14.2 months for SSC and 27.9 ± 14.2 months for ZC (*p* = 0.42). No significant differences were observed among the operators in the number of pulp treatments performed or in the quantity and type of crowns placed. The mean age of the patients at treatment was 5.1 ± 1.6 years (range 3–9). No statistically significant correlation was found between the age at the time of treatment and the development of the pathology (r = 0.077, *p* = 0.364). Additional analysis of failed cases showed no significant association between patient age and the likelihood of failure (*p* > 0.05).

Of the examined teeth: 22 (16%) were upper first primary molars, 13 (9%) were upper second primary molars, 63 (46%) were lower first primary molars and 40 (29%) were lower second primary molars. Statistically significant differences were not found of first or second primary molars, in the upper or lower jaw, with the development of any pathology (*p* = 0.993). Subgroup analyses confirmed no significant difference in pathology occurrence by tooth type (upper/lower, first/second molar). Table 1).

**Figure 1:**
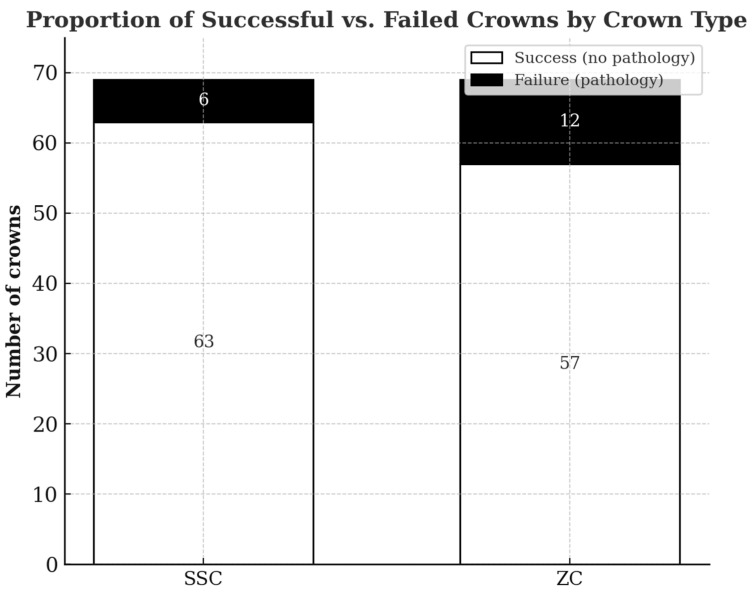
Proportion of successful vs. failed crowns by crown type (SSC vs. ZC). Proportion of successful vs. failed crowns by crown type (SSC vs. ZC). Abbreviations: SSC = Stainless Steel Crown; ZC = Zirconia Crown.

Of 138 crown treatments, 120 (87%) were categorized as successful and 18 as failures. Of the 120 successful crowns, 26 (19%) exfoliated naturally and 94 (68%) are still being followed. Of the 138 crowns examined, pathology was found in 18 (13%) (Table 1). Pathology developed in 9% (6/69) of the SSCs and 18% (12/69) of ZCs (*p* = 0.0025). Accordingly, of the 18 crowns that developed pathology, 12 (67%) were ZC and 6 (33%) were SSC. Of the 18 teeth that developed pathology, 12 (67%) presented resorption defects, 4 (22%) presented abscesses and 2 (11%) were extracted. The age of the child and the specific teeth that were treated were not found to correlate with the development of pathology (yes or no) (*p* = 0.855 and *p* = 0.093, respectively). Table 2 presents subgroup analysis by pulp status and type of therapy (pulpotomy, pulpectomy, none) was performed; no significant associations with pathology were found (*p* = 0.767 and *p* = 0.601). The distribution of successful vs. failed crowns by crown type is shown in Figure 1.

**Figure 2:**
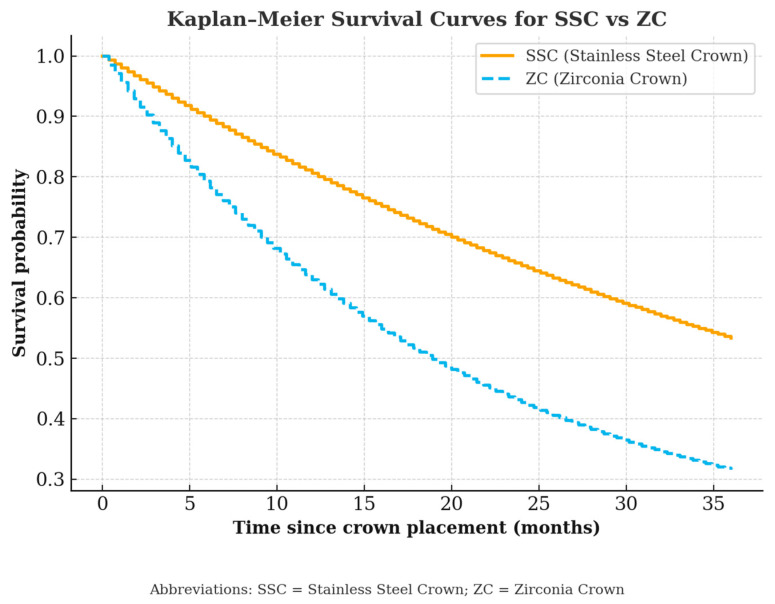
Kaplan–Meier survival curves comparing SSC vs. ZC. Kaplan–Meier survival curves comparing SSC vs. ZC.

Thirteen (9%) crowns showed lack of retention; of them, 11 (85%) were ZCs and 2 (15%) were SSCs (*p* = 0.009). Eighty-eight (64%) teeth underwent pulp treatment. The presence of pulp treatment and the type of pulp treatment (pulpotomy or pulpectomy) did not correlate with the development of pathology (*p* = 0.767 and 0.601, respectively). The time lapsed until the appearance of pathology was longer for SSCs than ZCs (29.09 ± 17.05 vs. 12.5 ± 6.91 months, *p* = 0.003). Survival time was longer for SSCs than ZCs (29.00 ± 15.66 vs. 25.15 ± 12.35 months, *p* = 0.034) (Table 3). No significant correlation was found between the patient’s age and the time of development of any pathology (r = 0.077, *p* = 0.364) (Figure 2).

## 5. Discussion

This retrospective study assessed the 2-year follow-up development of radicular pathology in primary molars restored with zirconia crowns (ZCs) or stainless steel crowns (SSCs), with a mean follow-up of approximately 27 months. Although both crown types demonstrated high overall success rates (87%), ZCs were associated with a significantly higher incidence of radicular pathology (18%) compared to SSCs (9%) (*p* = 0.0025). These findings are clinically relevant given the growing parental preference for esthetic restorations in pediatric dentistry [8,9]. ZCs have gained popularity due to their esthetics, biocompatibility, and favorable gingival response [10,11,12]. However, their clinical performance, particularly regarding retention and radicular outcomes, requires careful evaluation. In our study, retention loss was significantly more common in ZCs (16%) than in SSCs (3%) (*p* = 0.009). This aligns with prior studies [10,13], which attributed lower ZC retention to their passive fit and reliance on luting cement, in contrast to SSCs that benefit from mechanical retention via crimping [14]. This highlights an important difference in technique sensitivity. ZCs require precise tooth preparation, passive seating, and reliable cementation, while SSCs are more forgiving due to their ability to be crimped and adapted. Clinical success with ZCs therefore depends heavily on strict adherence to manufacturer instructions and adequate operator training.

The longer survival time of SSCs observed in our cohort is consistent with Agrawal et al. (2022) [15], who reported higher short-term retentivity in SSCs. ZCs require more extensive tooth preparation, which may increase the risk of pulpal insult and reduce marginal sealing potential. Despite using glass ionomer cement for all crowns in our study-considered optimal for ZC retention [16,17]—retention failures and subsequent pathology were still more frequent in the ZC group.

The choice of adhesive cement and isolation method is critical for zirconia crown success. Although glass ionomer is common, resin-modified or bioactive cements may enhance bonding, and strict moisture control (ideally with rubber dam) is essential. SSCs, by contrast, rely on mechanical retention and are less sensitive to these factors.

Margin design is another important factor: supragingival placement improves hygiene and retention, while subgingival margins may enhance esthetics but increase the risk of gingival inflammation. SSCs are more forgiving due to their crimpability and ability to adapt regardless of margin level.

Together, these differences explain why SSCs remain the more durable and forgiving restoration, particularly in complex cases or when optimal isolation cannot be achieved.

Previous systematic reviews have reported high overall ZC retention (up to 89%) but note significant variability, with some studies showing retention as low as 50%, largely influenced by the cement type [18,19] found that at least 2 mm of remaining tooth structure is necessary for adequate ZC retention. In our study, pathology developed sooner in ZCs than SSCs (12.5 vs. 29.1 months, *p* = 0.003), and overall survival was shorter for ZCs (*p* = 0.034).

Interestingly, we found no significant correlation between patient age, tooth type, or presence/type of pulp therapy and the development of pathology. This suggests that failures may be more attributable to mechanical or restorative factors, such as marginal adaptation and cementation quality. These findings support those of Seale and Randall (2015) [2] and Holsinger et al. (2021) [8], who emphasized the importance of final restoration quality over pulp status in predicting future success. This confirms that age was not a predictor of failure in this cohort.

Despite higher pathology rates in ZCs, both crown types exhibited high clinical success. This aligns with Ludovichett et al. (2021) [1], who concluded that SSCs remain the gold standard, particularly for high-risk cases, though ZCs are a viable alternative when esthetics are a primary concern.

While most existing studies report outcomes over short follow-up periods (6–12 months), our study contributes valuable long-term data (>24 months). Patnana et al. (2022) [5] and Pei & Chen (2022) [6] found comparable short-term success between ZCs and SSCs but may underestimate late-onset complications such as root resorption. Our results suggest that ZCs are more technique-sensitive and require ideal clinical conditions to achieve success.

ZCs have shown favorable gingival health outcomes in previous studies, but in our cohort this advantage was offset by higher pathology and retention loss [6]. Thus, ZCs may be better suited for anterior or esthetically sensitive cases, while SSCs may offer superior durability in posterior teeth or in patients with behavioral or hygiene challenges.

Our findings emphasize the importance of balancing esthetic benefits against clinical durability when selecting restorative options for primary molars. In cooperative patients and well-isolated clinical environments, ZCs remain a reliable option. However, for patients with poor oral hygiene, extensive pulpal involvement, or limited behavioral compliance, SSCs may yield more predictable outcomes. 18% pathology in ZCs vs. 9% in SSCs reflects a clinically meaningful difference. Esthetic benefits must be balanced with durability, parental satisfaction, and child quality-of-life considerations.

Enhanced training in ZC preparation and cementation, as well as the use of advanced adhesive materials, may help reduce failure rates. Regular radiographic follow-up beyond the first year is recommended to identify delayed complications.

This study has several limitations. Its retrospective design limits control over variables such as operator technique and documentation quality. The broad age range (3–9 years) and differences in sedation use could also have influenced outcomes. Two-dimensional radiographs may have missed early pathologic changes, and extracted teeth were not available for assessment of marginal integrity. Finally, although the average follow-up exceeded two years, longer-term data are still needed.

Future research should include randomized controlled trials comparing various ZC brands, luting agents (e.g., bioactive cements), and operator skill levels. Studies incorporating cost-effectiveness, patient satisfaction, and quality-of-life outcomes will further inform clinical decision-making.

## 6. Conclusions

While both crown types demonstrated high success, zirconia crowns require meticulous technique, proper isolation, and careful case selection. They are best suited for esthetically demanding cases with cooperative patients. Stainless steel crowns remain the more durable and predictable choice for posterior molars, particularly in patients with poor oral hygiene or high caries risk.

## Figures and Tables

**Table 1:** Failures of crown performance by tooth type (first vs. second molar; upper vs. lower).

Tooth Type	Number of Teeth	Pathology (*n*, %)	No Pathology (*n*, %)
Upper 1st molar	22	2 (9%)	20 (91%)
Upper 2nd molar	13	1 (8%)	12 (92%)
Lower 1st molar	63	9 (14%)	54 (86%)
Lower 2nd molar	40	6 (15%)	34 (85%)

Abbreviations: *n* = number; % = percentage.

**Table 2:** Subgroup analysis of pathology occurrence according to pulp therapy type (pulpotomy, pulpectomy, no pulp therapy).

Pulp Therapy Type	Number of Teeth	Pathology (*n*, %)	No Pathology (*n*, %)
None	50	6 (12%)	44 (88%)
Pulpotomy	70	8 (11%)	62 (89%)
Pulpectomy	18	4 (22%)	14 (78%)

Abbreviations: *n* = number; % = percentage.

**Table 3:** Comparison of Clinical Performance Between Stainless Steel Crowns (SSCs) and Zirconia Crowns (ZCs).

Crown Type	Time to Pathology Development (Months, Mean ± SD)	Survival Time (Months, Mean ± SD)	*p*-Values
SSC (*n* = 69)	29.09 ± 17.05	29.86 ± 15.66	0.003
ZC (*n* = 69)	12.50 ± 6.91	25.15 ± 12.35	0.034

Abbreviations: SSC = Stainless Steel Crown; ZC = Zirconia Crown; SD = Standard Deviation; Statistical comparisons performed using independent samples *t*-test. Significance defined as *p* < 0.05. Sample sizes: SSC (*n* = 69), ZC (*n* = 69).

## Data Availability

The data supporting the findings of this study are not publicly available due to patient privacy and ethical restrictions. Data may be obtained from the corresponding author upon reasonable request and with approval from the institutional ethics committee.
